# Expression Profiling of Glucosinolate Biosynthetic Genes in *Brassica oleracea* L*.* var. *capitata* Inbred Lines Reveals Their Association with Glucosinolate Content

**DOI:** 10.3390/molecules21060787

**Published:** 2016-06-17

**Authors:** Arif Hasan Khan Robin, Go-Eun Yi, Rawnak Laila, Kiwoung Yang, Jong-In Park, Hye Ran Kim, Ill-Sup Nou

**Affiliations:** 1 Department of Horticulture, Sunchon National University, Suncheon 540-950, Korea; gpb21bau@gmail.com (A.H.K.R.); yeege91@hanmail.net (G.-E.Y.); rawnak.2010@gmail.com (R.L.); ykw7685@naver.com (K.Y.); jipark@sunchon.ac.kr (J.-I.P.); 2 Plant Systems Engineering Research Center, Korea Research Institute of Bioscience and Biotechnology (KRIBB), 125 Gwahangno, Daejeon 34141, Korea; kimhr@kribb.re.kr

**Keywords:** glucosinolates, biosynthetic genes, expression analysis, *Brassica oleracea* var. *capitata*, genotypic variations

## Abstract

Glucosinolates are the biochemical compounds that provide defense to plants against pathogens and herbivores. In this study, the relative expression level of 48 glucosinolate biosynthesis genes was explored in four morphologically-different cabbage inbred lines by qPCR analysis. The content of aliphatic and indolic glucosinolate molecules present in those cabbage lines was also estimated by HPLC analysis. The possible association between glucosinolate accumulation and related gene expression level was explored by principal component analysis (PCA). The genotype-dependent variation in the relative expression level of different aliphatic and indolic glucosinolate biosynthesis genes is the novel result of this study. A total of eight different types of glucosinolates, including five aliphatic and three indolic glucosinolates, was detected in four cabbage lines. Three inbred lines BN3383, BN4059 and BN4072 had no glucoraphanin, sinigrin and gluconapin detected, but the inbred line BN3273 had these three aliphatic glucosinolate compounds. PCA revealed that a higher expression level of *ST5b* genes and lower expression of *GSL-OH* was associated with the accumulation of these three aliphatic glucosinolate compounds. PCA further revealed that comparatively higher accumulation of neoglucobrassicin in the inbred line, BN4072, was associated with a high level of expression of *MYB34* (Bol017062) and *CYP81F1* genes. The *Dof1* and *IQD1* genes probably trans-activated the genes related to biosynthesis of glucoerucin and methoxyglucobrassicin for their comparatively higher accumulation in the BN4059 and BN4072 lines compared to the other two lines, BN3273 and BN3383. A comparatively higher progoitrin level in BN3273 was probably associated with the higher expression level of the *GSL-OH* gene. The cabbage inbred line BN3383 accounted for the significantly higher relative expression level for the 12 genes out of 48, but this line had comparatively lower total glucosinolates detected compared to the other three cabbage lines. The reason for the genotypic variation in gene expression and glucosinolate accumulation is a subject of further investigation.

## 1. Introduction

Cabbage, one the most important vegetable crops throughout the world, is a member of the vegetable species *Brassica oleracea* that contains a wide variety of glucosinolates [1,2,3,4,5]. The subspecies *B. oleracea* var. *capitata* produced 12 different kinds of glucosinolates in its edible organ, including eight types of aliphatic, three types of indolic and one type of aromatic glucosinolates in the Korean cabbage varieties [5]. Cabbage is an important ingredient in many important Korean and Chinese dishes. This subspecies was found to produce glucoerucin, which was absent in the edible organs of kale, kohlrabi and cauliflower [5]. However, the number of glucosinolates present in cabbage was found to be variable in different studies [1,2,4,5]. This is probably because the content and type of glucosinolates in cabbage subspecies varies between growing season [2,6], anthocyanin content and leaf age [6] and also between tissue types: roots, shoots [4,7,8], *etc*. The content and varieties of glucosinolates also widely varies in the edible organ between different cultivars of this subspecies [1,2,9] probably because of the variation in tissue types. Despite a large number of studies reporting that glucosinolate content and types had intra-subspecific variation in cabbage varieties, the associated gene involved in such variation has not been well elucidated.

**Figure 1:**
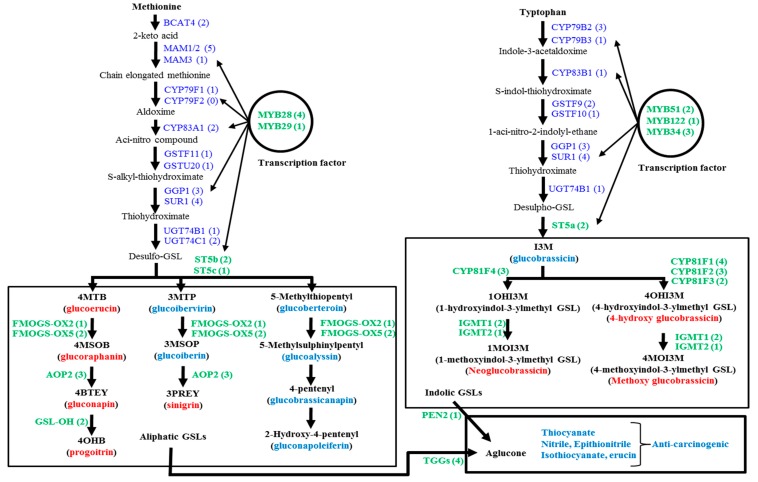
Metabolic pathways involved in the biosynthesis of aliphatic and indolic glucosinolates (GSL) after Liu *et al*. [10], along with the related genes of *B. oleracea*. The GSL molecules measured by HPLC are denoted as red bold (see Table S1 for their chemical structure names). The relative expression level of green bold genes was measured by qPCR. Numbers in parentheses denote the number of genes involved in this process (Adopted from Yi *et al*. [5]).

The biosynthetic pathways of glucosinolates and the break-down of these biomolecules follow species level variation, especially since these pathways are quite different in the vegetable species *B. oleracea* compared to those in *Arabidopsis* and *B. rapa*. *B. oleracea* has greater diversity compared to *B. rapa* and *B. napus* [10]. *B. oleracea* has about 84 glucosinolate biosynthesis genes and 22 catabolism-related genes [5,10]. A recent study explored two available web sources, Bolbase and EnesmblPlants, to compile the coding DNA sequences of glucosinolate biosynthesis-related genes and then catalogued reverse-transcript PCR-based semi-quantitative expression profiling of 84 genes in the edible organs of four *B. oleracea* subspecies, including cabbage leaf samples [5]. The authors reported that the biosynthesis of 12 diversified types of glucosinolates was not dependent on the expression of all 84 genes in the cabbage subspecies. Rather, the expression of one of a few genes (probably the key regulatory genes) in each step of glucosinolate biosynthesis might result in the accumulation of a particular glucosinolate in the edible organs of different *B. oleracea* subspecies [5]. Glucosinolate biosynthesis generally follows a characteristic three-step process. The process commences with the addition and elongation of side chains composed of methylene groups to the amino acid molecules. The second and third steps are the core structure formation and modification in the side chains of the elongated amino acid chain, respectively. The modification of side chains is accomplished by oxidation, hydroxylation, methoxylation, sulfation and glycosylation, processes that involve different genes [11,12,13]. The initial amino acid precursors of glucosinolate biosynthesis are generally methionine (or alanine, leucine, isoleucine and valine) for aliphatic glucosinolates and tryptophan and phenylalanine (or tyrosine), respectively, for indolic and aromatic glucosinolates (Figure 1; [13,14]).

In *Arabidopsis*, the amino acid side chain elongation begins by the action of methylthioalkylmalate synthase (*MAM*) in association with acid: sodium symporter family protein 5 (BASS5) and branched-chain aminotransferase (BCAT) at the beginning of glucosinolate biosynthesis [15,16,17,18]. The next step is the formation of the core structure, which is a five-step process, including: (i) aldoxime formation by cytochromes P450 of the *CYP79* and *CYP83* families; (ii) aldoxime oxidation to nitrile oxides or aci-nitro compounds by cytochromes P450 of the *CYP83* family; (iii) thiohydroximic acid formation by C-S cleavage; (iv) desulfoglucosinolate formation by *S*-glucosyltransferase; and (v) the formation of glucosinolates by sulfotransferase [19,20]. The desulfoglucosinolates undergoes secondary modifications to produce particular glucosinolate biomolecules that involve several gene loci, for example: *GS-OX*, *GS-AOP*, *GS-OH* are involved in aliphatic glucosinolate biosynthesis, and *CYP81*, *IGM* are involved in indolic glucosinolate biosynthesis (Figure 1). *MYB*-transcription factor-related genes trans-activate the functions of several genes involved in side chain elongation and core-structure formation (Figure 1; [21,22,23,24,25,26,27]). A few other transcription factor-related genes other than *MYB*, such as *TFL2*, *IQD1* and *Dof1.1*, also trans-activate both aliphatic and indolic glucosinolate biosynthesis [28,29,30,31]. In cabbage subspecies, expression profiling of none of those genes was studied to elucidate any intra-subspecies variation in gene expression. The present study therefore was conducted to explore the quantitative expression profiling of glucosinolate biosynthesis in four different cabbage inbred lines with variation in morphological appearance. The glucosinolates present in inbred lines were detected, and their contents were also quantified. An association between the glucosinolate biosynthesis-related gene and the glucosinolate content was explored.

## 2. Results and Discussion

### 2.1. Genotypic Variation in the Relative Expression Profiling of Glucosinolate Biosynthesis Genes

**Figure 2:**
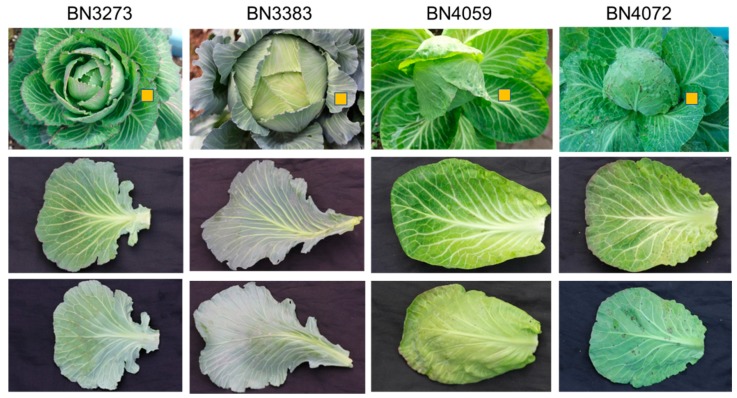
Genotypic variation in the leaf morphology of middle-aged leaves of four *B. oleracea capitata* inbred lines. Each column represents the whole plant, the upper- and lower-surface of an individual leaf, respectively. The leaves marked with orange-boxes were sampled. The inbred line BN3273 was the calibrator in the relative expression analysis.

**Figure 3:**
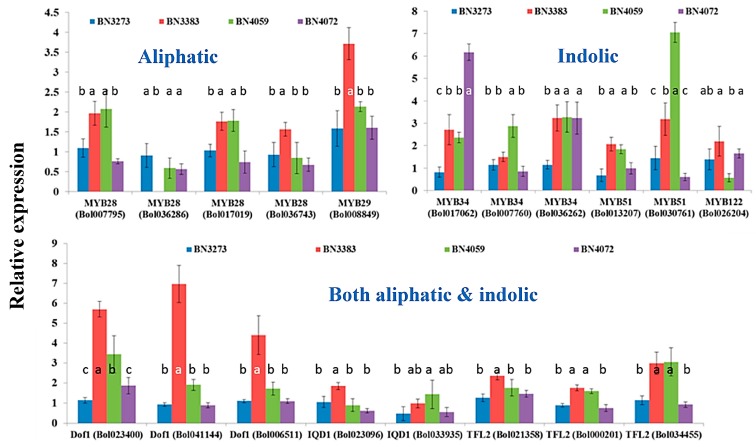
Relative expression level of transcription factor-related genes in four cabbage inbred lines involved in aliphatic, indolic and both aliphatic and indolic glucosinolate biosynthesis in *B. oleracea*. Vertical bars indicate the standard deviation of the means of nine observations. Different letters (a, b, c) indicate statistically significant difference between inbred lines.

This study investigated the relative expression level of a total of 48 genes related to glucosinolate biosynthesis in *B. oleracea* var. *capitata* in four inbred lines, BN3273, BN3383, BN4059 and BN4072 (Figure 2) with contrasting morphological variations (Figure 2). The external morphological variations include variation in leaf shape and appearance (Figure 2). Moreover, none of those four inbred lines had any common parents according to Asia Seed Company (Seoul, Korea). The color of the leaves of BN3273 and BN3383 was pale green, and that of BN4059 and BN4072 was green (Figure 2). While calculating the relative expression level of different genes, the inbred line BN3273 was considered as the control. The inbred line BN3383 accounted for a remarkably higher level of relative gene expression for the *MYB29* transcription factor-related gene associated with aliphatic glucosinolate biosynthesis compared to the other three lines (Figure 3, Table S2). *MYB28* accession Bol036286 recorded the lowest level of gene expression for the same line compared to the other three lines (Figure 3, Table S2). The inbred line BN4072 accounted for a comparatively lower expression level of *MYB28* accessions Bol007795 and Bol017019 and *MYB29* accession Bol008849 compared to BN4059 (Figure 3, Table S2).

The inbred line BN4072 accounted for, remarkably, more than a six-fold, higher level of gene expression for the indolic transcription factor-related gene *MYB34* accession Bol017062 compared to the control line BN3273 (Figure 3, Table S2). Another line BN4059 accounted for a seven-fold higher expression level compared to the control line BN3273 (Figure 3, Table S2). The same line had about a three-fold higher level of expression for the *MYB34* (Bol007760) gene (Figure 3, Table S2). The inbred line BN3383 had a comparatively higher level of expression for three *Dof1* genes associated with both aliphatic and indolic glucosinolate biosynthesis compared to the three other lines (Figure 3, Table S2). Another transcription factor-related gene associated with both aliphatic and indolic glucosinolate biosynthesis, *TFL2* accession Bol034445, was highly expressed in both BN3383 and BN4059 lines compared to the other two cabbage inbred lines (Figure 3, Table S2).

**Figure 4:**
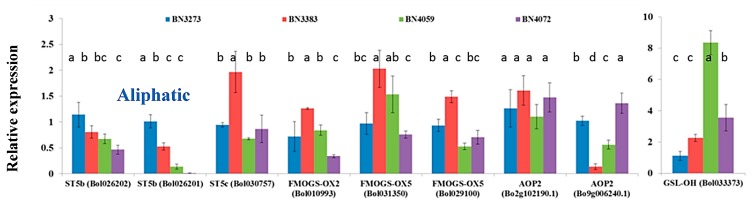
Relative expression level of aliphatic glucosinolate biosynthesis-related genes in four cabbage inbred lines of *B. oleracea*. Vertical bars indicate the standard deviation of the means of nine observations. Different letters (a, b, c) indicate statistically significant difference between inbred lines.

The inbred line BN3383 accounted for a high level of gene expression up to two-fold for the following aliphatic glucosinolate biosynthesis genes: *ST5c* accession Bol030757, *FMOGS-OX2* accession Bol010993, *FMOGS-OX5* accessions Bol031350 and Bol029100; compared to the control line BN3273 (Figure 4, Table S2). Inbred lines BN4059 and BN4072 accounted for a strikingly lower expression level of *ST5b* accession Bol026201 compared to the other two lines, BN3383 and BN3273 (Figure 4, Table S2). The gene *AOP2* (Bol9g006240.1) had more than a seven-fold lower level of relative gene expression in the inbred line BN3383 compared to the control line BN3273 (Figure 4, Table S2). Another aliphatic transcription factor-related gene *GSL-OH* (Bol033373) had more than an eight-fold higher expression level in the inbred line BN4059 (Figure 4, Table S2).

**Figure 5:**
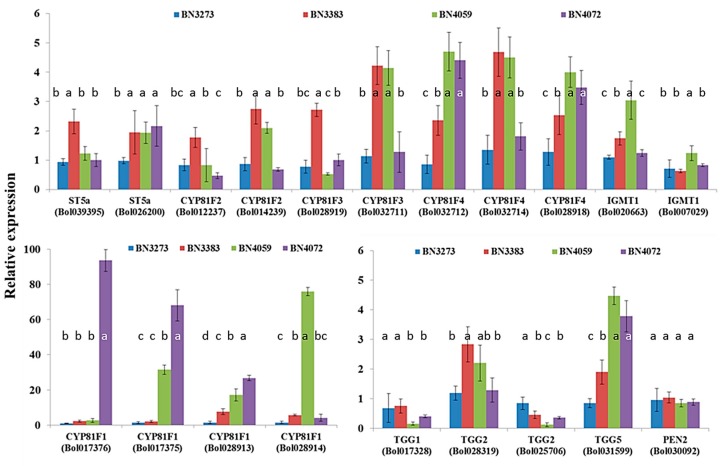
Relative expression level of indolic glucosinolate biosynthesis- and break-down-related genes in four cabbage inbred lines of *B. oleracea.* Vertical bars indicate the standard deviation of the means of nine observations. Different letters (a, b, c) indicate statistically significant difference between inbred lines.

Four indolic biosynthesis-related gene accessions, namely *ST5a* (Bol039395), *CYP81F2* (Bol012237), *CYP81F2* (Bol014239) and *CYP81F3* (Bol028919), accounted for approximately a two-fold higher level of gene expression for the cabbage line BN3383 compared to the control line BN3273 (Figure 5, Table S2). The inbred line BN4059 coupled with BN3383 accounted for approximately a four-fold higher expression level of the *CYP81F3* (Bol028919) and *CYP81F4* (Bol032714) genes compared to the two other cabbage lines (Figure 5, Table S2). The relative expression level of *CYP81F4* (Bol032712) and *CYP81F4* (Bol028918) was significantly higher in the BN4059 and BN4072 lines compared to the other two inbred lines (Figure 5, Table S2). The line BN4072 accounted for more than a 90-fold and a 60-fold higher level of gene expression of *CYP81F1* accessions Bol017376 and Bol017375, respectively, compared to the control line BN3273 (Figure 5, Table S2). Another cabbage line, BN4059, accounted for the highest expression level of *CYP81F1* (Bol028914) gene, about 80-fold compared to the three other lines (Figure 5). The relative expression level of *CYP81F1* accessions Bol017376 and Bol0128913 was comparatively higher in cabbage lines BN4059 and BN4072 compared to BN3273 and BN3383 (Figure 5, Table S2).

The glucosinolate break-down-related gene *TGG5* (Bol031599) was expressed highly in the inbred lines BN4059 and BN4072 compared to BN3273 and BN3383 (Figure 5, Table S2). The level of expression of two other break-down-related genes, *TGG1* (Bol017328) and *TGG2* (Bol025706) in the BN4059 and BN4072 lines, was remarkably low compared to the BN3273 and BN3383 lines (Figure 5, Table S2).

### 2.2. Genotypic Variation in Glucosinolate Contents

**Figure 6:**
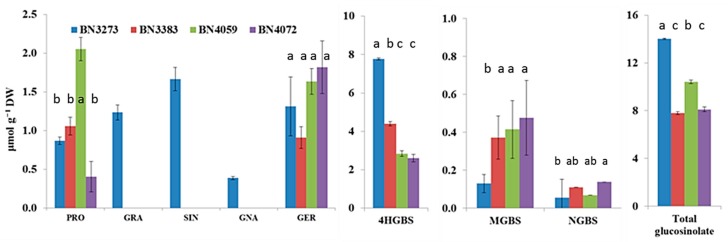
Contents of aliphatic and indolic glucosinolates produced in four inbred lines of cabbage. Vertical bars indicate the standard error of the means of nine observations. The response factors for glucosinolate quantification were obtained from European Economic Community [32], International Standards Organization [33] and American Oil Chemists Society [34]. Different letters (a, b, c) indicate statistically significant difference between inbred lines. PRO, progoitrin; GRA, glucoraphanin; SIN, sinigrin; GNA, gluconapin; GER, glucoerucin; 4HGBS, 4-hydroxy-glucobrassicin; MGBS, methoxy-glucobrassicin and NGBS, neoglucobrassicin.

HPLC analysis detected a total of eight types of glucosinolate molecules in four cabbage inbred lines (Figure 6). Those were progoitrin, glucoraphanin, sinigrin, gluconapin, glucoerucin, 4-hydroxy-glucobrassicin (4HGBS), methoxy-glucobrassicin (MGBS) and neoglucobrassicin (NGBS). However, three aliphatic glucosinolates, glucoraphanin, sinigrin and gluconapin, were detected only in the cabbage line BN3273 (Figure 6). The same line also had comparatively higher amounts of 4HGBS and total glucosinolate, but lower MGBS compared to the other three lines (Figure 6, Table S3). Only two other aliphatic glucosinolates, progoitrin and glucoerucin, were detected in all four lines (Figure 6). 4HGBS, MGBS and NGBS are the three indolic glucosinolates that were detected in all four inbred lines (Figure 6). Unlike the relative expression level of a notable number of genes, the cabbage inbred line BN3383 did not have a higher amount of glucosinolates compared to the other three lines (Figure 6). The inbred line BN4059 had the highest content of progoitrin (Figure 6, Table S3), whereas BN4072 had the highest content of NGBS (Figure 6).

### 2.3. Association between Glucosinolate Content and Gene Expression

#### 2.3.1. Association between Aliphatic Glucosinolate Content and Transcription Factor-Related Genes

The association between glucosinolate content and the relative gene expression level was explored through principal component analysis (PCA). In principal component 1 (PC1), the aliphatic transcription factor-related genes and glucosinolate content accounted for a negative contrast (Table 1). The coefficient for *MYB* genes was positive (except Bol036286), and those for glucoraphanin, sinigrin, gluconapin and glucoerucin content were negative (Table 1). This contrast is largely explained by the significant genotypic variation between the BN3383, BN4059, BN4072 lines and the BN3273 line, as only the BN3273 line contained glucoraphanin, sinigrin and gluconapin (Table 1). PC2 accounted for 23.3% of the data variation and estimated a larger contrast between BN4059, BN4072 and BN3273, BN3383 for a higher content of glucoraphanin, sinigrin and gluconapin, a lower content of glucoerucin and vice versa in association with the expression level of *MYB* genes (Table 1). The PC3 explaining 15.3% of the data variation accounted for a positive association between higher progoitrin content in line BN4059 and a higher expression level of *MYB28* accession Bol007795 (Table 1).

#### 2.3.2. Association between Indolic Glucosinolate Content and Transcription Factor-Related Genes

PC1 obtained from a PCA for the indolic glucosinolate content and transcription factor-related genes accounted for 34.3% of the data variation, but the coefficients of this PC were dominated by the higher expression of *MYB34* (Bol007760) and *MYB51* (Bol030761) genes in BN4059 and BN3383 inbred lines compared to the other two genotypes (Table 2). PC2 accounted for a positive association between NGBS content and the relative expression level of the *MYB34* (Bol017062) gene (Table 2). This contrast is largely explained by the higher NGBS content and simultaneously higher expression level of *MYB34* (Bol017062) gene in the BN4059 line compared to BN3273 (Table 2). PC2 in Table S4 indicated a notable contrast between the BN4059, BN4072 lines and the BN3273 and BN3383 lines for contrasting higher and lower content of glucoerucin and MGBS, respectively (Figure 6). This variation was largely explained by the contrasting expression level of the *Dof1* (Bol041144), *Dof1* (Bol006511) and *IQD1* (Bol023096) genes (Table S4). Part of this variation is also explained by the absence of glucoraphanin, sinigrin and gluconapin in the BN4059 and BN4072 lines (Table S4).

#### 2.3.3. Association between Aliphatic Glucosinolate Content and Biosynthesis-Related Genes

PC1 accounted for a positive association among the higher level of expression of *ST5b* genes and the lower level of expression of *GSL-OH* (Bol033373) in BN3273 and their association with the content of glucoraphanin, sinigrin and gluconapin (Table 3). PC2, which explained 31.8% of the data variation, was largely influenced by the higher level of expression of the *ST5c* and *FMOGS-OX* genes in BN3383 (Table 3). PC3 accounted for 17.1% of the data variation, indicating that higher progoitrin accumulation in the line BN4059 might be associated with the higher expression level of *GSL-OH* (Bol033373) and the lower expression level of the *AOP2* (Bo2g102190) gene compared to the BN4072 line (Table 3, Figure 4 and Figure 6).

#### 2.3.4. Association between Indolic Glucosinolate Content and Biosynthesis-Related Genes

PC1 in another PCA between indolic glucosinolate content explained 35.1% of the data variation (Table S5). This PC highlighted a contrast in the expression level of most of the indolic biosynthesis-related genes in BN3273 (generally having a lower expression level) compared to the other three lines (Figure 5, Table S5). BN3273 had a comparatively lower expression level for all indolic biosynthesis-related genes compared to the other three lines (Figure 5). The variation explained by PC2 was dominated by the higher expression level of *CYP81F1* accessions Bol017376, Bol017375 and Bol028913 and the lower expression level of *CYP81F2*, *CYP81F2* and ST5a (Bol026200) in BN4072 compared to the other two lines, BN3273 and BN4072 (Figure 5, Table S5). The variation in PC2 is largely accounted for by the strikingly higher expression of level of *CYP81F1* genes in the inbred line BN4072 (Table S5). PC3, explaining 16.6% of the data variation, is dominated by the higher NGBS content in BN4072 and BN3383 compared to the other two lines (Table S5).

### 2.4. Discussion

The present study investigated the relative gene expression level of 48 genes from the aliphatic and indolic metabolic pathways of glucosinolate biosynthesis and also estimated the content of glucosinolate in four inbred lines of cabbage with contrasting morphological variation (Figure 2). Only one out of four genotypes, BN3273, had three aliphatic glucosinolates detected, glucoraphanin, sinigrin and gluconapin. The cabbage inbred lines BN3383, BN4059 and BN4072 had none of those three aliphatic glucosinolates. This genotype-specific variation was associated with the expression level of some key genes. PCA analysis between the expression level of aliphatic glucosinolate contents and the relative expression level of aliphatic glucosinolate biosynthesis-related genes indicated that the existence of glucoraphanin, sinigrin and gluconapin might be associated with the comparatively higher level of expression of *ST5b* genes and the lower expression level of *GSL-OH* genes in BN3273 (Table 3, Figure 4). Thus, in the inbred lines BN3383, BN4059 and BN4072, the non-existence of glucoraphanin, sinigrin and gluconapin might be associated with the lower level of expression of the *ST5b* genes (Table 3, Figure 4). The non-existence of those three aliphatic glucosinolates in the BN4059 and BN4072 lines might also be associated with the level of transactivation of *ST5b* genes by the following transcription factor-related genes: *MYB28* (Bol007795), *MYB28* (Bol017019), *MYB28* (Bol036743) and *MYB29* (Bol008849) (Table 1). Such transactivation of *ST5b* genes by the *MYB* transcription factors has been discussed by Variyar *et al*. [35].

Two separate PCs involving indolic transcription factor-related and biosynthesis genes (PC2 in Table 2 and Table S5, respectively) indicated that a notably higher expression level of the *MYB34* (Bol017062) gene (Figure 3) is associated with trans-activation of the expression of the *CYP81F1* (Bol017375, Bol017376, Bol028913) genes (Figure 5) in the inbred line BN4072 for a comparatively higher accumulation of MGBS and NGBS in that particular line (Figure 6). On the other hand, the strikingly higher level of expression of *CYP81F1* (Bol028914) and two *IGMT1* genes was associated with comparatively lower content of NGBS in another line, BN4059 (Table S5).

The HPLC analysis in this study only detected eight types of glucosinolates; those included no aromatic glucosinolate compound (Figure 6). In our previous study, we detected a total of 12 glucosinolates, including glucoiberverin (GIV), glucoiberin (GIB), glucobrassicanapin (GBN), glucoraphanin (GRE), glucobrassicin (GBS) and gluconasturtiin (GST, an aromatic glucosinolate), in cabbage subspecies [5]. None of those six glucosinolates were detected in this study. However, in this study, we detected 4HGBS, which is a very close intermediate glucosinolate compound of GBS. The reason behind this variation might be related to the genetic variations of different cabbage genotypes, environmental variation or may be partly related to the HPLC procedure.

A high level of gene expression of the *TGG2* (Bol028319) break-down-related gene in the cabbage inbred line BN3383 suggested that break-down of glucosinolate molecules is also high in that inbred line compared to the other three lines (Figure 5). In contrast, the lowest level of expression of *TGG1* (Bol017328) and *TGG2* (Bol025706) in inbred lines BN4059 and BN4072 suggested a lower level of break-down of glucosinolate molecules compared to the two other inbred lines, although a contrasting expression level was observed for the *TGG5* gene (Figure 5). The lower level of expression of break-down-related genes might be associated with the lower level of the defense capacity of the BN4059 and BN4072 lines against herbivory and microbial attack.

One of the notable observations in this study is that out of a the total of 48 genes, 12 genes accounted for a significantly higher level of gene expression in a particular line, BN3383, compared to the other three cabbage lines (Figure 2, Figure 3, Figure 4 and Figure 5). However, none of the eight detected glucosinolate molecules were higher in content in BN3383 compared to the other three lines (Figure 6). The reason behind such variation is a subject of further investigation. However, a lower level of glucosinolate in that particular line might be associated with enhanced glucosinolate break-down through the elevated expression of *TGG2* genes (Figure 5). Moreover, sequence variants are another possible explanation, and intuitively, the other includes various protein levels due to post-transcriptional modifications and various levels of enzymatic activities regulated at the post-translational level, which would be specific for the line BN3383.

The general role of the constitutive glucosinolate molecules is providing defense to plants from insects and microbes by their anti-oxidative properties [36,37,38,39]. A notable number of previous studies confirmed that the content of glucosinolates varies between genotypes of cabbage [1,2,3,4]. However, none of those previous studies explored the genetic background of such variation. The reason behind the higher or lower level of gene expression in different cabbage genotypes and associated variation in glucosinolate accumulation is a subject of further variation. Intuitively, there might have been variation in the functional component within the gene sequence, including the presence of the InDel (insertion or deletion) in the base sequence, single nucleotide polymorphism, *etc*., and also there may be variation in enzymatic activities regulated at the post-translational level.

## 3. Experimental Section

### 3.1. Plant Materials and Growth Conditions

A total of four inbred lines with contrasting morphological variation from the cabbage (*B. oleracea* var. *capitata*) subspecies were selected for this study (Figure 2). Seeds were obtained from Asia Seed Co., Ltd. (Seoul, Korea). The seeds were sown and seedlings were raised in a garden soil mixture in a culture room. Seedlings were transferred at four weeks of age to a glasshouse. The plants were allowed to grow for another three months before collecting leaf samples for estimating glucosinolates and also for quantifying the relative expression of glucosinolate biosynthetic genes. Leaf samples used for both real-time PCR and metabolite analysis were collected from the middle-aged leaves (see Figure 2).

### 3.2. Primer Design for Glucosinolate Biosynthesis Genes

As the objective of this study was to investigate the genotypic variation in the expression level of glucosinolate biosynthetic genes related to identified aliphatic and indolic glucosinolates (Figure 6), therefore, a total of 48 genes related to transcription and biosynthesis of glucosinolate were selected from the two respective biosynthetic pathways for relative expression analysis (Figure 1, Table 4). These 48 genes included all transcription factor-related genes associated with side chain elongation and core structure formation and biosynthetic genes that convert crude desulfoglucosinolates to a particular aliphatic or indolic glucosinolate. Among them, five and six genes respectively were aliphatic and indolic transcription factor-related and 10 and 15 genes were aliphatic and indolic glucosinolate biosynthesis related genes, respectively. The other eight genes from the *Dof*, *IQD* and *TFL* subfamilies were also transcription factor related to both pathways. In addition, these 48 genes also included five glucosinolate break-down-related genes. To design primers for each gene, Primer3 web-based software, Primer3web version 4.0.0 (http://primer3.ut.ee/, Table 4), was used. The primer specificity of the genes was initially checked by using the primer-blast option in NCBI (http://www.ncbi.nlm.nih.gov/tools/primer-blast/) and also by using the similarity search option in the Bolbase database (http://www.ocri-genomics.org/bolbase/blast/blast.html). As a further confirmatory analysis, the sequences of all highly homologous genes and the designed primers were aligned using ClustalW software [40], and both forward and reverse sequences of the primers of the target gene were obtained from the dissimilar regions of the homologues. To test the efficiency of the primers, the pooled cDNA of equal concentrations, 300 ng·µL^−1^, of four cabbage inbred lines was serially diluted by 10-fold, and 1.0 µL of the 10^0^, 10^−1^, 10^−2^, 10^−3^, 10^−4^ and 10^−5^ diluted cDNA was used as the template in each reaction with forward and reverse primers. The qRT-PCR reaction was performed for 40 cycles along with the melting curve in triplicate along with no template control. A semi-log plot for C_t_ versus fold dilution was drawn to find out the slope. The efficiency of the primer was calculated by using the following formula, e = 10^^(−1/slope)^. A primer with −3.321928 slopes was found 100% efficient. Primers with an 85%–100% efficiency level were chosen for expression analysis, otherwise redesigned to obtain the efficiency within the expected range.

### 3.3. cDNA Synthesis and Real-Time Quantitative PCR Analysis

An RNeasy mini kit (Catalogue No. 74106, Qiagen, Valencia, CA, USA) was used to extract the total RNA of the leaf samples. cDNA was synthesized from the total RNA of each sample using a PrimeScript-based kit (Takara Bio, Inc., Shiga, Japan). iTaq™ SYBR^®^ Green Super-mix was used with ROX (ROX is a positive reference dye) (Foster City, CA, USA) for real-time PCR. The reaction volume was 20 µL, where 1 µL cDNA of each sample was used. The concentration of cDNA of all genotypes was diluted from 60–70 ng·µL^−1^. The targeted DNA segment was amplified by denaturation at 95 °C for 10 min, followed by 40 cycles of amplification with denaturation at 94 °C for 20 s, annealing at 58 °C for 20 s and a final incubation and signal acquisition at 72 °C for 25 s. For some genes, the annealing temperature was increased from 58 °C–63 °C to improve the melting peaks. The fluorescence was recorded for each sample, while Light Cycler 96 software was used for quantification (Cq) analysis (Roche, Germany). There were three biological replicates for each line, and each biological replicate was repeated three times. The relative expression level was calculated by the comparative 2^−ΔΔCt^ method [41]. Three different *actin* genes, *actin1*, *actin2* and *actin3*, GenBank Accession Nos. AF044573 [42], JQ435879 [43] and XM_013753106 [44], respectively, which were expressed in all inbred lines, were the reference (Table 4 and Table S6, Figure S1). cDNA of four cabbage lines was diluted to 60, 120 and 180 ng·µL^−1^ concentrations to test the expression efficiency of the three reference genes. Reference genes were found quite stable under three different cDNA concentrations (Figure S2). The expression stability of all genes, including three reference genes, was calculated using NormFinder software (see the Supplementary excel data file) [45]. All genes accounted for the low intra-group (within genotype) variation, but inter-group variation was high for some notable genes that accounted for significant genotypic variation, for example *CYP81F1, ST5b* (Bol026201) and *MYB28* (Bol036286), suggesting that the expression of the genes was valid. The average Cq value of these three *actin* genes was used for calculating the relative expression of each glucosinolate biosynthesis-related gene. While calculating the relative expression level of the each glucosinolate biosynthesis gene, the cabbage inbred line BN3273 was considered as the calibrator. The relative expression of the other three lines for each glucosinolate biosynthesis-related gene was then accounted for comparing to the expression level of BN3273.

### 3.4. Desulfoglucosinolate Extraction for HPLC Analysis

The modified HPLC protocol of Choi *et al*. [6] was used to extract desulfoglucosinolates from the leaf samples. About 10 g of frozen tissue were ground after treatment with 70% ethyl alcohol. The ground samples were incubated initially at 70 °C for 10 min followed by incubation at room temperature for 1 h. Then, the samples were centrifuged at 10,000× *g* for 8 min at 4 °C to precipitate the tissues and proteins. The supernatant was collected to conduct anion-exchange chromatography. This centrifugation process was repeated twice, and the supernatants from three steps were pooled in a 5-mL tube. The pooled supernatants contained crude glucosinolates. The crude glucosinolate solution was then mixed with 0.5 mL 50 mM barium acetate and 0.5 mL 50 mM lead acetate. The solution was then passed through a desulfation process. In this process, the solution was centrifuged once more at 2000× *g* for 10 min before loading into a pre-equilibrated column. In this column, the crude glucosinolate sample was rinsed with distilled water several times. The desulfation commenced after the addition of 250 μL aryl sulfatase and continued for 16 h. The desulfated solution was eluted with 1 mL distilled water. To remove any impurities in the desulfated glucosinolates, the eluted solution was centrifuged at 20,000× *g* for 4 min at 4 °C and filtered through a PTFE filter (13 mm, 0.2 μm, Advantec, Pleasanton, CA, USA). The desulfoglucosinolate samples were then analyzed in a Waters 2695 HPLC system (Waters, Milford, MA, USA) equipped with a C_18_ column (Zorbax Eclipse XBD C_18_, 4.6 mm × 150 mm, Agilent Technologies, Palo Alto, CA, USA). The mobile phase solvents used in the HPLC system were water and acetonitrile. The flow of desulfoglucosinolates into the system was 0.4 mL min^−1^ at room temperature. A PDA 996 UV-visible detector (Waters) was used to detect the desulfoglucosinolates at a wavelength of 229 nm. The detected glucosinolates were quantified by comparison with a standard curve prepared of commercial sinigrin. Mass spectrometry analysis (HPLC/MS, Agilent 1200 series, Agilent Technologies) facilitated the identification of individual glucosinolate molecules (HPLC/MS, Agilent 1200 series, Agilent Technologies).

### 3.5. Statistical Analysis

Analysis of variance (ANOVA) for the relative expression level of each gene and glucosinolate content against four different genotypes was analyzed via the one-way command using MINITAB 17 statistical software (Minitab Inc., 1829 Pine Hall Rd, State College, PA, USA). Tukey’s pairwise comparison was conducted for mean separation. A PCA was conducted taking either aliphatic or indolic glucosinolate content with the corresponding relative expression level of biosynthesis genes as a set of variables. PC scores obtained under four cabbage lines were also analyzed using one-way ANOVA.

## 4. Conclusions

The present study investigated the relative expression level of 48 genes related to glucosinolate biosynthesis and also estimated glucosinolate content in four cabbage inbred lines with contrasting morphological variation. The expression level of the majority of genes and glucosinolate content varied significantly in a genotype-dependent manner. A total of eight glucosinolates were detected in four inbred lines with significant variation in their content among them. Only one inbred line BN3273 had glucoraphanin, sinigrin and gluconapin detected. The biosynthesis and accumulation of these three aliphatic glucosinolates were associated with the variable expression level of *ST5b* and *GSL-OH* genes that were trans-activated by the *MYB28* and *MYB29* genes. For the indolic glucosinolates, higher accumulation of NGBS in the line BN4072 was associated with a higher level of expression of the *MYB34* and *CYP81F1* genes. The relative expression level of *ST5b* (Bol026201), *TGG1* (Bol017328) and *TGG2* (Bol025706) was consistently lower in the BN4059 and BN4072 lines compared to the other two inbred lines. A total of 12 genes were highly expressed in the inbred line BN3383, but that particular line had the least content of total glucosinolates measured. The possible reason for such genotypic variation is a matter of further investigation. However, the candidate genes with a contrasting level of relative expression might be cloned and sequenced to find out any possible existence of single nucleotide polymorphism, and any variation in enzymatic activities regulated at the post-translational level might be explored.

## Figures and Tables

**Table 1:** Principal component analysis for aliphatic glucosinolate contents and the relative expression level of transcription factor-related genes in four cabbage lines of *B. oleracea.* PC, principal component; *p*, statistical significance; SD, standard deviation.

Variable	PC1	PC2	PC3
Progoitrin	0.18	−0.16	0.69
Glucoraphanin	−0.37	−0.38	−0.01
Sinigrin	−0.37	−0.38	−0.01
Gluconapin	−0.33	−0.33	−0.02
Glucoerucin	−0.11	0.40	0.37
*MYB28* (Bol007795)	0.34	−0.29	0.36
*MYB28* (Bol036286)	−0.36	−0.11	0.26
*MYB28* (Bol017019)	0.33	−0.31	0.19
*MYB28* (Bol036743)	0.22	−0.43	−0.27
*MYB29* (Bol008849)	0.37	−0.20	−0.27
%variation explained	47.2	23.3	15.3
*p* (genotype)	<0.001	<0.001	0.001
Genotype	Mean PC Scores (±SD)		
BN3273	−2.92 ± 0.43	−1.31 ± 1.32	−0.03 ± 0.31
BN3383	2.55 ± 0.16	−0.85 ± 0.27	−0.99 ± 0.49
BN4059	1.24 ± 0.29	0.06 ± 0.52	1.66 ± 0.90
BN4072	−0.87 ± 0.16	2.01 ± 0.66	−0.69 ± 0.91

**Table 2:** Principal component analysis for indolic glucosinolate contents and the relative expression level of transcription factor-related genes in four cabbage lines of *B. oleracea.* PC, principal component; *p*, statistical significance; SD, standard deviation.

Variable	PC1	PC2
4HGBS	−0.24	−0.29
MGBS	0.13	0.26
NGBS	−0.11	0.46
*MYB34* (Bol017062)	−0.06	0.53
*MYB34* (Bol007760)	0.52	−0.15
*MYB34* (Bol036262)	0.31	0.43
*MYB51* (Bol013207)	0.41	0.19
*MYB51* (Bol030761)	0.54	−0.14
*MYB122* (Bol015939)	−0.28	0.28
%variation explained	34.3	31.0
*p* (genotype)	<0.001	<0.001
Genotype	Mean PC Scores (±SD)	
BN3273	−1.71 ± 0.23	−2.17 ± 0.38
BN3383	0.25 ± 0.20	0.86 ± 0.51
BN4059	2.64 ± 0.44	−0.63 ± 0.23
BN4072	−1.17 ± 0.17	1.94 ± 0.81

**Table 3:** Principal component analysis for aliphatic glucosinolate contents and the relative expression level of the biosynthesis-related genes in four cabbage lines of *B. oleracea.* PC, principal component; *p*, statistical significance; SD, standard deviation.

Variable	PC1	PC2	PC3
Progoitrin	−0.16	0.09	0.55
Glucoraphanin	0.42	−0.08	0.17
Sinigrin	0.42	−0.07	0.17
Gluconapin	0.42	−0.09	0.14
Glucoerucin	−0.07	−0.24	0.05
*ST5b* (Bol026201)	0.34	0.09	0.12
*ST5b* (Bol026202)	0.41	0.15	0.12
*ST5c* (Bol030757)	0.03	0.41	−0.21
*FMOGS-OX2* (Bol010993)	0.01	0.44	0.18
*FMOGS-OX5* (Bol029100)	−0.13	0.40	0.18
*FMOGS-OX5* (Bol031350)	0.13	0.40	−0.21
*AOP2* (Bo2g102190)	0.01	0.10	−0.52
*AOP2* (Bo9g006240)	0.09	−0.42	−0.19
*GSL-OH* (Bol033373)	−0.34	−0.09	0.35
%variation explained	36.3	31.8	17.1
*p* (genotype)	<0.001	<0.001	<0.001
Genotype	Mean PC Scores (±SD)		
BN3273	3.58 ± 0.41	−0.69 ± 0.61	0.57 ± 0.74
BN3383	−0.32 ± 0.35	3.29 ± 0.55	−0.80 ± 0.53
BN4059	−2.09 ± 0.38	−0.46 ± 0.18	1.88 ± 0.75
BN4072	−1.17 ± 0.28	−2.13 ± 0.18	−1.65 ± 0.95

**Table 4:** Primer sequences of 48 glucosinolate biosynthetic-related genes used for the relative expression analysis of four cabbage inbred lines with morphological variation through qPCR.

Gene Name	Accession Number	cDNA Size (bp)	Forward Primer Sequence	Reverse Primer Sequence	Product Size (bp)
Transcription factor-related genes (19 genes)					
Aliphatic					
*MYB28*	Bol007795	558	CCACACCAGTTCAGAGAGGT	GGGAAATGGATCGAAGTCAGC	221
	Bol036286	615	GAAGGTAGCTTGAATGCTAATAC	ATTCATGTAGTGCTCCTCATTC	249
	Bol017019	426	GTTGCGGCTAAGGTCACTTCT	CAGAAGTAGCGTTGATCTCATGC	223
	Bol036743	426	CTTGGGCGCTGCTACATTAC	ATCGTTCTCCTCGTTGTGGT	241
*MYB29*	Bol008849	513	CGCCCAAGACTTCTGAGTT	TGATATTGCCCATGGAAGCTG	234
Indolic					
*MYB34*	Bol007760	843	TGAAGGAGGATGGCGTACTC	CAGTTCGTCCCGCCAAATTA	203
	Bol017062	951	AAGGTGGATGGCGTACTCTC	TGTGAGTGGTTGGATCGACA	279
	Bol036262	294	CCCCGAGTTCTTTAGCAACC	TCCAAGTCCAGATCGTCTTCT	198
*MYB51*	Bol013207	1002	CCAGAGATTCCAGAGAAGC	CAAGTCACACTGCTACTACTAC	233
	Bol030761	990	CAGACAACTATATCGAGTAACG	TATCATTAACGGTCATCTGG	274
*MYB122*	Bol026204	981	GACCATTCCGAGACATTGCC	GCATCGTGGATCATGTGGAG	284
Both aliphatic and indolic					
*Dof1.1*	Bol023400	1005	GACGAAACATAGCAGCTCCG	ACCGGGTTGTTCTTCCATCT	227
*Dof1.1*	Bol041144		TTGGTCACAGCCTACGAACT	ACTTGGTGATGAGGGAGTCG	260
*Dof1.1*	Bol006511		ACCAACTTCCACCTTTCCCA	AAGCTGCTCTCATTCTCGGT	163
*IQD1*	Bol023096		TGTCCTCGATGCAACTCCAT	ACCGAGAATGAGAGCAGCTT	287
*IQD1*	Bol033935		AAGCTGCTCTCATTCTCGGT	CCTCTTTGGTTGCCTTGGTC	195
*TFL2*	Bol021358	1146	ACGATGCTGCTGAGAAGTCT	CCTGGTCCCCTTAACTCGTT	199
*TFL2*	Bol000201		AGCGGGAAAAGAAGTGGAGA	GCCTTTCCTCTTTCCCTCCT	166
*TFL2*	Bol034455		AAGAGGGAGAAGCAGCCATT	AACCTCCACTGGCTTGATGA	239
Aliphatic biosynthesis-related genes (9 genes)					
*ST5b*	Bol026201	1035	CCGAGCCGTCAGAATTCAAG	GCTATGGCGAAAGTGAGAGC	247
	Bol026202	1035	AAGCCTTGACTTTCGCCATC	ACTTCACAACTGAGTCCGGT	204
*ST5c*	Bol030757	1014	CCACGCCCAAAACTTCTTCA	TGAGTGGAGAAGAGCGTGTT	246
*FMOGS-OX2*	Bol010993	1386	GAGAAGGTATCCGAGCCACA	GTCCACTGCAAACAACGACT	200
*FMOGS-OX5*	Bol029100	1347	CTTGCTCCAACGCTTTCCTT	CCTCAGCTCTCCAGTGTTCA	280
	Bol031350	1380	GACACTACACAGAGCCTCGT	CCCCGGGAAGCTTCTCATAT	234
*AOP2*	Bo2g102190		GGAACGTGTCTCCAAAACCC	TAGCACCATCACCAGCATCA	354
	Bo9g006240		CCAGGAAGTGAGAAGTGGGT	TAGCACCATCACCAGCATCA	517
*GSL-OH*	Bol033373	243	GATTGTGCAAAAGGCTTGT	AGAGCATTAGGATTAGGAGGA	188
Indolic biosynthesis-related genes (15 genes)					
*ST5a*	Bol026200	1017	GTCCGGTTGCAAGATGGTTT	CCTCTCCGGGTTCTCTTTGT	214
	Bol039395	1014	TGCCGTTTGTGAAGAGGTTG	CCCAATCTCCAACCTTCCCT	210
*CYP81F4*	Bol032712	1506	CGGTGGAGGAGAAGGAGAAA	CTGACACATGGCTCGTAACG	226
	Bol032714	960	ACCCTGGTGAATACTTGCCA	GAAACACACTGAAGCAGAAC	239
	Bol028918	1503	GTTTGCGGCATCAGAGACAT	GAATAGTCCACGCGTTCACC	299
*CYP81F1*	Bol017375	369	AAGCAGAGCGGTTCAAGAAG	GCGTGACCATTGTGTTACCA	204
	Bol017376	246	CCGTCTCCTTCAACGGTTCT	CGACGTATTTACCGGTGAGC	170
	Bol028913	1500	GAGACCTCCGCAGTAACCTT	GTCCTCCGTCGGTCTTCTAG	222
	Bol028914	1497	CTTTCCAACTGACGGCCAAA	CGTTAGGTCCGAGAAAAGCG	257
*CYP81F2*	Bol012237	933	GCAGCCGTGACACTAGAATG	TCCGCCAATCTTGAGGTCTT	231
	Bol014239	1482	TTGTACCGCGTTCTCCTTCT	GACACCATCCTCTGACCCAA	238
*CYP81F3*	Bol028919	1500	TAACAGCGGAGGAGAAGACG	CACCTTCTAACTGGGCCTGA	260
	Bol032711	1492	CCGTCTCACCAACTTCCTCT	CTTCTCAAAGCTCCCTCCCA	292
*IGMT1*	Bol007029	1119	GTGTTCCTCTCACCTTCCGA	GTGTTGAGGAAGACGCTGTC	260
	Bol020663	342	AGATGCCATGATCTTGAAACGT	CCAGCAATGATAAGCCTGACA	298
Glucosinolate break-down-related genes (5 genes)					
*TGG1*	Bol017328	822	TCTTAACGTGTGGGATGGCT	CCTCCTTTGTTCACTCCCCT	210
*TGG2*	Bol028319	1179	TCGTCTCAACAGTAGCAGCT	AGTAGCGTTGAGTTCGTCCA	220
	Bol025706	663	GGTGAGTAGGGGAGTGAACC	TTCCTCGGTGAAGTTGGGAA	244
*TGG5*	Bol031599	1326	CCAGATCACAGTTCCGGAGA	ACTATACGCCGGCTCAAGAA	293
*PEN2*	Bol030092	1299	GCATCATCATCCAACAGCGT	ACGCCTTGATCAGTTCTCCA	207
House-keeping genes					
*actin1*	AF044573		TTCTCTCTTCCACACGCCAT	CTTGTCCTGCGGGTAATTCG	235
*cctin2*	JQ435879		GTCGCTATTCAAGCTGTTCTCT	GAGAGCTTCTCCTTGATGTCTC	251
*actin3*	XM_013753106		ATCACACTTTCTACAATGAGC	TCGTAGATTGGCACAGTGTGAG	241

## Supplementary Materials

Supplementary materials can be accessed at: http://www.mdpi.com/1420-3049/21/6/787/s1.

## Abbreviations

4HGBS: 4-hydroxyglucobrassicin
MGBS: methoxyglucobrassicin
NGBS: neoglucobrassicin
