# Supplementary material for: Expression Profiling of Glucosinolate Biosynthetic Genes in Brassica oleracea L. var. capitata Inbred Lines Reveals Their Association with Glucosinolate Content
Source: Molecules. 2016 Jun 17;21(6):787. doi: 10.3390/molecules21060787 (PMC6273970; doi:10.3390/molecules21060787)
Supplement: Supplementary file 1 [file molecules-21-00787-s001.zip › molecules-131375-supplementary-final/molecules-131375-supplementary-done.pdf]

Supplementary Materials: Expression Profiling of Glucosinolate Biosynthetic Genes in *Brassica Oleracea L.* var. *Capitata* Inbred Lines Reveals Their Association with Glucosinolate Content

Arif Hasan Khan Robin, Go-eun Yi, Rawnak Laila, Kiwoung Yang, Jong-In Park, HyeRan Kim and Ill-Sup Nou

Table S1. A list of glucosinolate compounds and their intermediaries with their chemical structure names, trivial names and different abbreviations used.

Common Name	Chemical Name	Abbreviations
Glucoerucin (GER)	4-(methylsulfanyl)butyl glucosinolate	4MTB
Glucoraphanin (GRA)	4-(methylsulfinyl)butyl glucosinolate	4MSOB
Sinigrin (SIN)	allyl glucosinolate	3PREY
Gluconapin (GNA)	3-butenylglucosinolate	3BTEY
Progoitrin (PRO)	4-hydroxybutyl glucosinolate	4OHB
4-hydroxy-glucobrassicin (4HGBS)	4-hydroxy 3-Indolylmethyl glucosinolate	4OHI3M
Neoglucobrassicin (NGBS)	1-methoxy-3-indolylmethyl glucosinolate	1MOI3M
4-methoxy Glucobrassicin (MGBS)	4-methoxy-3-indolylmethyl glucosinolate	4MOI3M

Table S2. *p* values indicating statistically significant variation between four cabbage lines in glucosinolate biosynthesis related gene expression level.

Gene Name	Accession Number	*p* (Genotype)
Transcription factor related genes (19 genes)		
MYB28	Bol007795	<0.001
	Bol036286	<0.001
	Bol017019	<0.001
	Bol036743	0.003
MYB29	Bol008849	<0.001
MYB34	Bol007760	<0.001
	Bol017062	0.001
	Bol036262	<0.001
MYB51	Bol013207	<0.001
	Bol030761	<0.001
MYB122	Bol026204	0.001
Dof1.1	Bol023400	<0.001
Dof1.1	Bol041144	<0.001
Dof1.1	Bol006511	<0.001
IQD1	Bol023096	<0.001
IQD1	Bol033935	0.027
TFL2	Bol021358	<0.001
TFL2	Bol000201	<0.001
TFL2	Bol034455	<0.001

Table S2. *Cont.*

**Gene Name**	**Accession Number**	***p* (Genotype)**
Aliphatic biosynthesis related genes (10 genes)		
ST5b	Bol026201	<0.001
	Bol026202	<0.001
ST5c	Bol030757	<0.001
FMOGS-OX2	Bol010993	<0.001
FMOGS-OX5	Bol029100	<0.001
	Bol031350	<0.001
AOP2	Bo2g102190	0.126
	Bo9g006240	<0.001
GSL-OH	Bol033373	<0.001
Indolic biosynthesis related genes (15 genes)		
ST5a	Bol026200	<0.001
	Bol039395	0.037
CYP81F4	Bol032712	<0.001
	Bol032714	<0.001
	Bol028918	0.026
CYP81F1	Bol017375	<0.001
	Bol017376	<0.001
	Bol028913	0.007
	Bol028914	<0.001
CYP81F2	Bol012237	0.001
	Bol014239	<0.001
CYP81F3	Bol028919	<0.001
	Bol032711	<0.001
IGMT1	Bol007029	0.005
	Bol020663	<0.001
Glucosinolate breakdown related genes (2 genes)		
TGG1	Bol017328	<0.001
TGG2	Bol028319	<0.001
	Bol025706	0.005
TGG5	Bol031599	0.002
PEN2	Bol030092	0.66

Table S3. *p* values indicating statistically significant variation between four cabbage lines for aliphatic and indolic glucosinolate contents.

**Glucosinolate**	***p* (genotype)**
Progoitrin	<0.001
Glucoraphanin	<0.001
Sinigrin	<0.001
Gluconapin	<0.001
Glucoerucin	0.113
4HGBS	0.006
MGBS	0.029
NGBS	0.003
Total	0.014

Table S4. Principal Component analysis for aliphatic and indolic glucosinolate contents and relative expression level of transcription-factor related genes (both aliphatic and indolic) in four cabbage lines of *B. oleracea*. PC, principal component; *p*, statistical significance.

Variable	PC1	PC2	PC3
Progoitrin	0.16	−0.10	0.54
Glucoraphanin	−0.28	0.27	0.21
Sinigrin	−0.27	0.28	0.21
Gluconapin	−0.28	0.26	0.20
Glucoerucin	−0.08	−0.34	0.14
Unidentified	0.07	−0.44	0.21
4HGBS	−0.14	0.12	0.17
MGBS	0.12	−0.33	−0.05
NGBS	0.08	−0.19	−0.43
Dof1 (Bol023400)	0.35	0.10	0.004
Dof1 (Bol041144)	0.30	0.25	−0.12
Dof1 (Bol006511)	0.29	0.25	−0.12
IQD1 (Bol023096)	0.19	0.36	−0.08
IQD1 (Bol033935)	0.23	−0.10	0.38
TFL2 (Bol021358)	0.32	0.09	−0.03
TFL2 (Bol000201)	0.32	0.12	0.22
TFL2 (Bol034455)	0.31	0.08	0.27
%variation explained	43.8	22.3	13.2
p (genotype)	<0.001	<0.001	<0.001
Genotype	Mean PC scores (±Sd)		
BN3273	−3.51 ± 0.39	1.66 ± 0.85	0.75 ± 0.43
BN3383	3.32 ± 0.34	1.69 ± 0.96	−0.76 ± 0.13
BN4059	1.43 ± 0.78	−1.31 ± 0.72	1.67 ± 1.03
BN4072	−1.24 ± 0.62	−2.04 ± 1.19	−1.66 ± 1.02

Table S5. Principal component analysis for indolic glucosinolate contents and relative expression level of biosynthesis related genes in four cabbage lines of *B. oleracea*. PC, principal component; *p*, statistical significance.

Variable	PC1	PC2	PC3
4HGBS	−0.22	−0.08	−0.04
MGBS	0.18	−0.10	0.16
NGBS	0.08	−0.14	0.45
ST5a (Bol026200)	0.14	0.30	0.30
ST5a (Bol039395)	0.28	−0.11	0.24
CYP81F1 (Bol017376)	0.01	−0.36	0.26
CYP81F1 (Bol017375)	0.15	−0.37	0.07
CYP81F1 (Bol028913)	0.21	−0.32	0.13
CYP81F1 (Bol028914)	0.30	0.01	−0.37
CYP81F2 (Bol012237)	0.13	0.36	0.07
CYP81F2 (Bol014239)	0.24	0.32	0.06
CYP81F3 (Bol032711)	0.02	0.25	0.44
CYP81F3 (Bol028919)	0.30	0.24	0.02
CYP81F4 (Bol032712)	0.33	−0.21	−0.02
CYP81F4 (Bol032714)	0.30	0.24	0.05
CYP81F4 (Bol028918)	0.36	−0.15	0.02
IGMT1 (Bol007029)	0.33	0.07	−0.27

Table S5. *Cont.*

Variable	PC1	PC2	PC3
IGMT1 (Bol020663)	0.25	−0.08	−0.35
% variation explained	35.1	32.5	16.6
p (genotype)	<0.001	<0.001	<0.001
Genotype		Mean PC scores (±Sd)	
BN3273	−3.58 ± 0.18	0.51 ± 0.28	−1.16 ± 0.32
BN3383	0.55 ± 0.77	2.91 ± 0.78	1.86 ± 0.32
BN4059	2.99 ± 0.65	0.11 ± 0.39	−1.95 ± 0.27
BN4072	0.03 ± 0.98	−3.53 ± 0.06	1.25 ± 1.05

Table S6. Ct values of three different *actin* genes. Each data is the average of two idendepent observations.

Genotype	Biological Replicate	Technical Replicate	Actin 1	Actin 2	Actin 3	Average
BN3273	1	1	15.2	17.5	15.2	15.9
	1	2	15.2	17.5	15.2	15.9
	1	3	15.2	17.5	15.2	15.9
	2	1	16.9	18.6	15.4	17.0
	2	2	16.9	18.6	15.4	17.0
	2	3	16.9	18.6	15.4	17.0
BN3383	1	1	17.7	20.7	15.2	17.8
	1	2	17.7	20.7	15.2	17.8
	1	3	17.7	20.7	15.2	17.8
	2	1	17.6	20.5	15.2	17.7
	2	2	17.6	20.5	15.2	17.7
	2	3	17.6	20.5	15.2	17.7
BN4059	1	1	18.5	17.7	16.5	17.5
	1	2	18.5	17.7	16.5	17.5
	1	3	18.5	17.7	16.5	17.5
	2	1	20.1	19.6	16.8	18.8
	2	2	20.1	19.6	16.8	18.8
	2	3	20.1	19.6	16.8	18.8
BN4072	1	1	18.8	17.7	13.7	16.7
	1	2	18.1	17.2	13.6	16.3
	1	3	18.1	17.2	13.6	16.3
	2	1	18.1	17.2	13.6	16.3
	2	2	18.1	17.2	13.6	16.3
	2	3	18.1	17.2	13.6	16.3

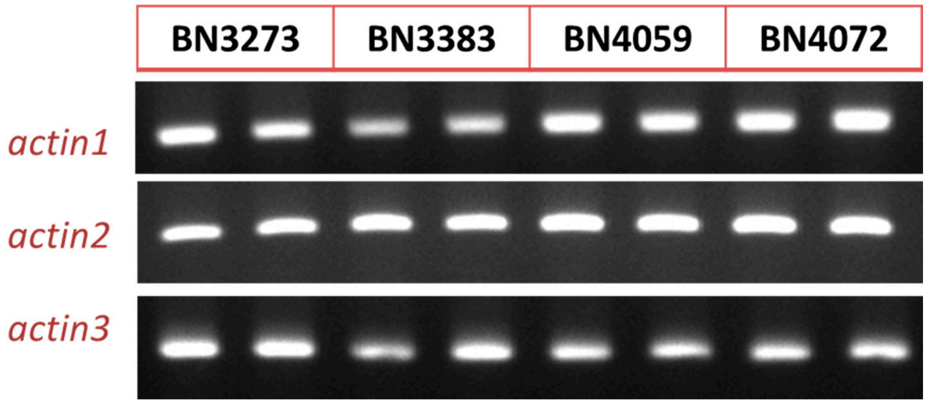

Figure S1. RT-PCR expression level of three actin genes in four cabbage lines.

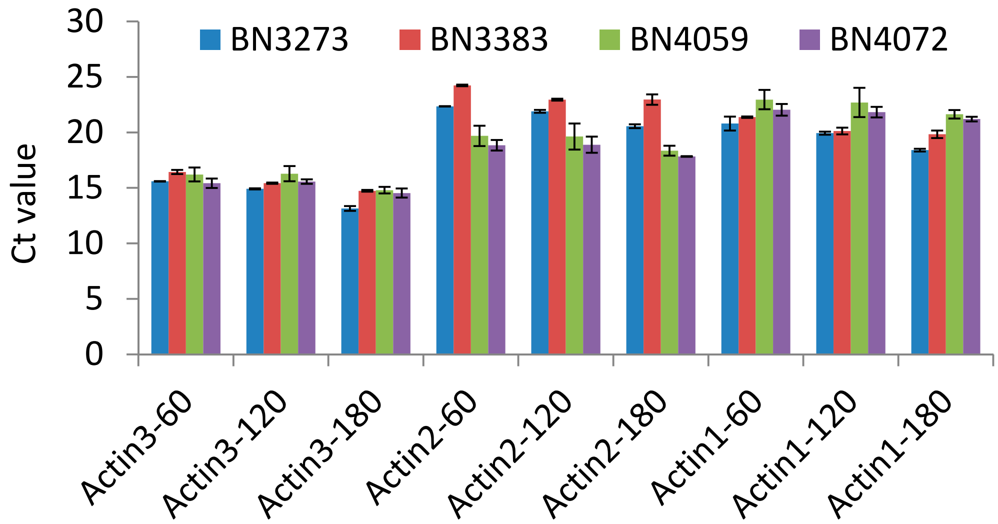

Figure S2. The expression level (Ct values) of three reference genes in response to three different concentrations of cDNA (60, 120 and 180 ng μL^{-1}) of four genotypes.
